# Large influence of atmospheric vapor pressure deficit on ecosystem production efficiency

**DOI:** 10.1038/s41467-022-29009-w

**Published:** 2022-03-29

**Authors:** Haibo Lu, Zhangcai Qin, Shangrong Lin, Xiuzhi Chen, Baozhang Chen, Bin He, Jing Wei, Wenping Yuan

**Affiliations:** 1 School of Atmospheric Sciences, Southern Marine Science and Engineering Guangdong Laboratory (Zhuhai), Sun Yat‐sen University, Zhuhai, Guangdong 519082 China; 2 School of Remote Sensing and Geomatics Engineering, Nanjing University of Information Science and Technology, Nanjing, Jiangsu 210044 China; 3 State Key Laboratory of Resource and Environmental Information System, Institute of Geographic Sciences and Natural Resources Research, Chinese Academy of Sciences, Beijing, 100101 China; 4 State Key Laboratory of Earth Surface Processes and Resource Ecology, College of Global Change and Earth System Science, Beijing Normal University, Beijing, 100875 China

**Keywords:** Carbon cycle, Carbon cycle

**arising from** Liu et al. *Nature Communications* 10.1038/s41467-020-18631-1 (2020)

Disentangling the respective effects of soil moisture (SM) and vapor pressure deficit (VPD) on ecosystem production is challenging but essential for understanding terrestrial carbon uptake in response to dryness stress. Recently, Liu et al.^1^ used solar-induced chlorophyll fluorescence (SIF) to indicate the ecosystem production and arrived at a conclusion that SM controls ecosystem production over larger global vegetated areas (71.3%) than VPD (26.7%). However, by further eliminating the coincident impacts of photosynthetically active radiation (PAR) and fraction of photosynthetically active radiation absorbed by plants (fPAR) on VPD and SM, using eddy covariance (EC) towers-based observations and global modeling data, we show that VPD rather than SM dominates ecosystem production efficiency over more flux sites and larger areas globally. Our analyses implicate that the impacts of VPD-induced atmospheric dryness on ecosystem production are at least equally, if not more important than SM, and the roles of VPD and SM should be fairly valued in ecosystem modeling.

The findings of Liu et al.^1^ are appealing, they developed an effective method to quantify the relative effects of VPD and SM on ecosystem production. However, it is loosely based on SIF which was entangled with the coincident changes of PAR and fPAR with VPD and SM, although Liu et al.^1^ tried to exclude the impacts of other environmental variables by limiting the data to a narrow temperature range and relatively high VPD and radiation. This leads to the inadequate reflection of VPD contributions to ecosystem production. Thus, we have reasons to question the use of SIF as dependent variable and that the coincident changes of PAR and fPAR with VPD and SM could interfere the analyses on VPD vs. SM effects. Contrary to SIF, fluorescence quantum yield (SIF_yield_) and light use efficiency (LUE) isolate the coincident changes of PAR and fPAR^2–4^, and can well represent the capability of ecosystem production. In the following analysis, we quantified the respective effects of VPD and SM on ecosystem production efficiency (i.e., SIF_yield_, LUE), our analysis provided an alternative perspective to Liu et al.^1^ that the importance and significance of both VPD and SM in ecosystem production efficiency need to be addressed.

To disentangle the respective effects of SM and VPD on ecosystem production, Liu et al.^1^ binned the satellite-based SIF observations into 10 bins of either SM or VPD. At each SM bin, the differences in SIF between the maximum and the minimum VPD bins (∆SIF(VPD|SM)) were used to indicate the VPD stress on SIF excluding the impacts of SM. Likewise, the differences in SIF between the minimum and the maximum SM at each VPD bin (ΔSIF(SM|VPD)) quantified the SM stress on SIF. It should be noticed that both ∆SIF(VPD|SM) and ΔSIF(SM|VPD) showed their respective restrictions of VPD and SM stress to SIF. To exclude the impacts of other environmental variables, Liu et al.^1^ used the observations only when (1) the daily mean temperature >15 °C, (2) daily average VPD >0.5 kPa, and (3) daily average photosynthetic photon flux density >500 µmol m^−2^ s^−1^. However, this approach did not entirely exclude the impacts of PAR and fPAR on SIF. We examined the differences of PAR and fPAR between the minimum and the maximum SM gradients at VPD bin (ΔPAR(SM|VPD) and ΔfPAR(SM|VPD)), and those between the maximum and the minimum VPD gradients at SM bin (∆PAR(VPD|SM)) and ∆fPAR(VPD|SM)). Figure 1 shows the positive ∆PAR(VPD|SM) across almost all study areas, implying the increasing PAR with the rising VPD at the SM bins (Fig. 1a, c). Coincident increasing PAR with VPD will benefit the ecosystem production and counteract the restrictions of rising VPD on ecosystem production. For the VPD bins, with decreasing SM, fPAR decreases over 83.46% of study areas, showing negative ΔfPAR(SM|VPD) (Fig. 1e, f). Coincident decreasing fPAR with SM gradients also reduces SIF, but this was not considered in the analysis of the impacts of SM on SIF.

**Fig. 1:**
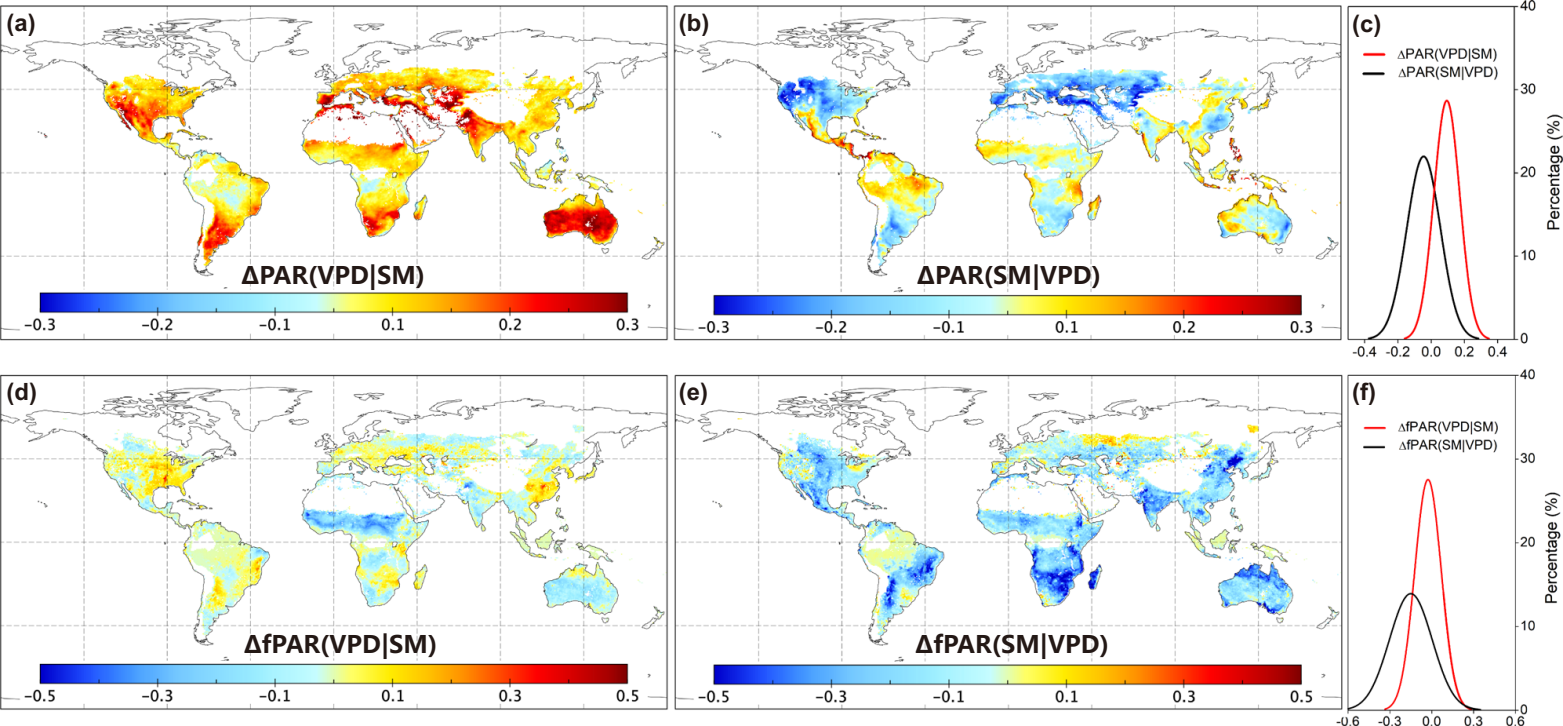
Effects of soil moisture (SM) and vapor pressure deficit (VPD) on photosynthetically active radiation (PAR) and fraction of photosynthetically active radiation absorbed by plants (fPAR) globally. **a**, **b** Indicate the spatial distribution of the changes in PAR caused by high VPD (ΔPAR(VPD|SM)) and low soil moisture (SM) (ΔPAR(SM|VPD)), and **c** shows the probability density function of ΔPAR. **d**–**f** Indicate the corresponding changes of fPAR. For better comparability in space, the PAR and fPAR data time series were normalized by the average exceeding 90th percentile per pixel. The units refer to the fractions relative to average PAR and fPAR exceeding the 90th percentile in each grid cell. Regions with sparse vegetation and regions without valid data are masked in white.

Following the method of Liu et al.^1^, we calculated the differences of SIF_yield_ between the minimum and the maximum SM gradients at VPD bin (ΔSIF_yield_(SM|VPD)) and the differences of SIF_yield_ between the maximum and the minimum VPD gradients at SM bin (∆SIF_yield_(VPD|SM)). The results show that SM only plays a dominant role over 44.37% of vegetated areas with valid data (Supplementary Fig. 1c), much smaller than Liu et al.^1^’s estimates of 71.30% of study area. These results question the robustness of the conclusion of Liu et al.^1^ if PAR and fPAR impacts were appropriately taken into consideration.

Relative to satellite-based SIF, worldwide EC towers-based ecosystem gross primary production (GPP) provides much more solid and direct evidence for benchmarking ecosystem productivity^5,6^. Here, we used the same method proposed by Liu et al.^1^ to distinguish the impacts of SM and VPD based on the estimated GPP from long-term flux tower observations at 40 sites (over 15 years) (Supplementary Table 1). LUE is used as an indicator of ecosystem production capability to isolate the coincident effects of PAR and fPAR^3,4^. The results show larger impacts of VPD on LUE than SM at 70% of the sites (Fig. 2a), and on average, the restrictions of VPD to LUE are 6.6 times than those of SM over all investigated sites (Supplementary Fig. 2). Further using global FLUXCOM GPP dataset simulated by machine learning models, we identified that on average, the ΔLUE(VPD|SM) is larger than ΔLUE(SM|VPD) over 58.89% of the study areas, implying relatively greater impact of VPD than SM on LUE (Fig. 2b and Supplementary Fig. 3).

**Fig. 2:**
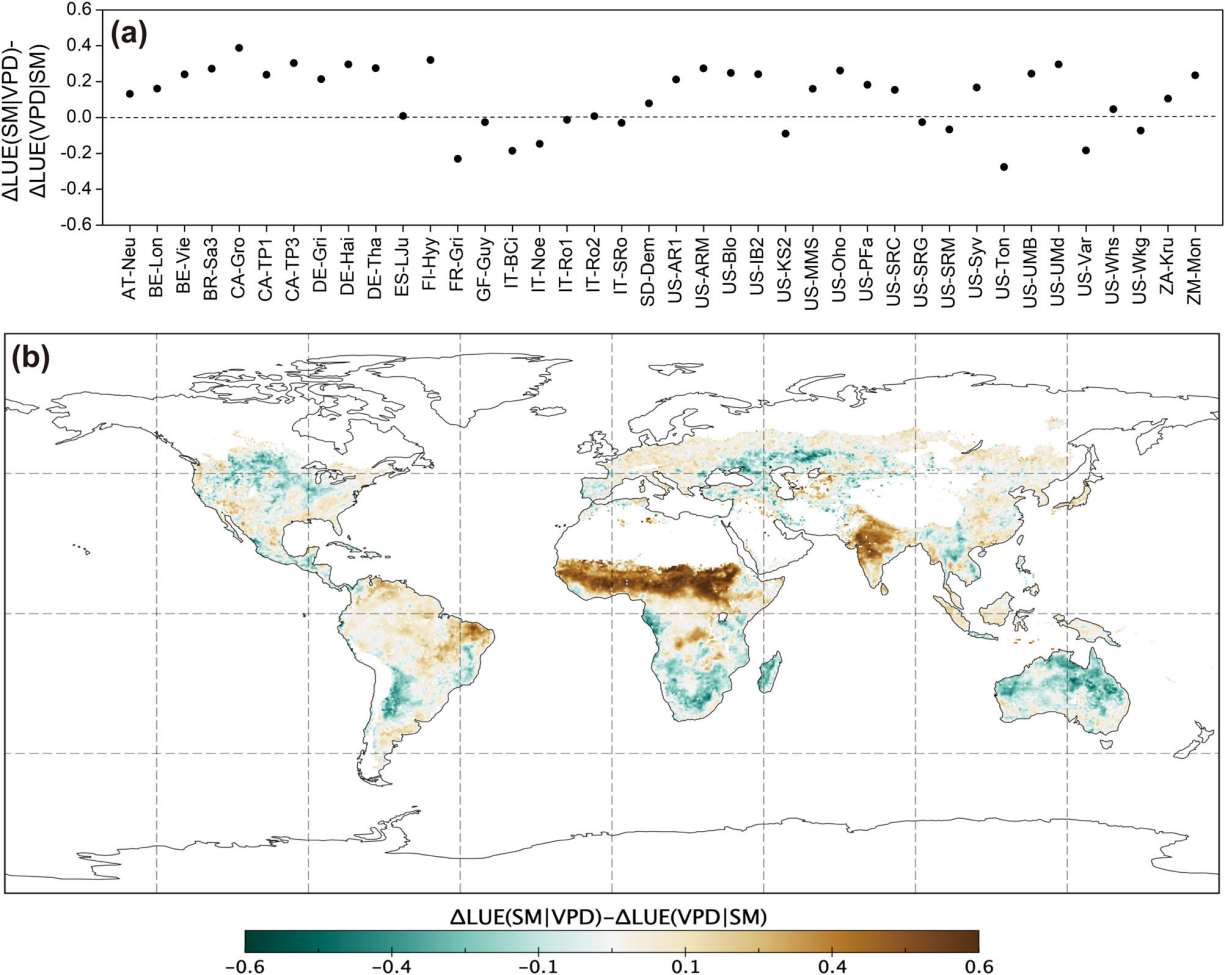
The comparison on impacts of soil moisture (SM) and vapor pressure deficit (VPD) on ecosystem light use efficiency (LUE) based on measurements of eddy covariance sites and global model data. **a** Differences between ΔLUE(SM|VPD) and ΔLUE(VPD|SM) at 40 eddy covariance sites. **b** Differences between ΔLUE(SM|VPD) and ΔLUE(VPD|SM) based on FLUXCOM dataset. The positive values indicate larger impacts of VPD relative to SM in **a**, **b**. Note, where ΔLUE(SM|VPD)>0, the difference equals to ΔLUE(VPD|SM) in **a**, **b**; where ΔLUE(VPD|SM)>0, the difference is ΔLUE(SM|VPD); and where both are positive, the difference is not shown. For better comparability in space, the LUE data time series was normalized by the average LUE exceeding 90th percentile. The units refer to the fractions relative to average LUE exceeding the 90th percentile for each eddy covariance site and each grid cell.

Recent studies highlighted the substantial changes of VPD and SM globally with the climate warming that may profoundly impact ecosystem production and carbon uptake^7–9^. The conclusion regarding to the relative contributions of VPD and SM on ecosystem production is very important for understanding responses of ecosystem production to dryness stress and reducing prediction uncertainties of terrestrial carbon uptake^10,11^. Liu et al.^1^ proposed an effective method to quantify the respective effects of VPD and SM on ecosystem production indicated by SIF globally. Differently, in this study we examined the respective impacts of VPD and SM on ecosystem production efficiency (i.e., SIF_yield_, LUE) excluding the coincident effects of PAR and fPAR with VPD and SM. Our analyses highlight larger and wider impacts of VPD on ecosystem production efficiency than SM. The role of VPD in ecosystem production is indispensable and should not be undervalued in order to appropriately model ecosystem responses to future climate conditions. Both the analyses from Liu et al.^1^ and this study are equally valid approaches to the same question. Our analyses offer an alternative perspective to Liu et al.^1^, and provide further insights into the internal component processes of ecosystem production in response to the effects of VPD and SM.

## Methods

This analysis followed the method of Liu et al.^1^ to quantify the respective impacts of SM and VPD on ecosystem production. To isolate the coincident impacts of PAR and fPAR on ecosystem production, we use fluorescence quantum yield (SIF_yield_) and light use efficiency (LUE) instead of SIF used by Liu et al.^1^, to better indicate the capacity of vegetation production^3,12^:

1 $${{{{{{\rm{SIF}}}}}}}_{{{{{{\rm{yield}}}}}}}=\frac{{{{{{\rm{SIF}}}}}}}{{{{{{\rm{PAR}}}}}}\,\times \,{{{{{\rm{fPAR}}}}}}}$$

2 $${{{{{\rm{LUE}}}}}}=\frac{{{{{{\rm{GPP}}}}}}}{{{{{{\rm{PAR}}}}}}\,\times \,{{{{{\rm{fPAR}}}}}}}$$

where PAR and fPAR indicate photosynthetically active radiation and fraction of photosynthetically active radiation respectively. LUE was calculated at both site-scale using EC measurements from 40 sites and global scale using global model data (i.e., FLUXCOM). The same data selection criterion of Liu et al.^1^ is followed at global scale, i.e., only SM or VPD bins where >10 data points are available. At site-scale, the threshold for LUE is set to 5 due to limited data availability. All datasets are listed in Supplementary Table 2.

## Supplementary information

Supplementary Information

## Data Availability

All data sources are given in Supplementary Information.

## Code availability

The primary code supporting the conclusion of this study are free to the public for scientific purposes and can be downloaded at 10.6084/m9.figshare.14528640.v1.
