# Supplementary material for: Large influence of atmospheric vapor pressure deficit on ecosystem production efficiency
Source: Nat Commun. 2022 Mar 29;13:1653. doi: 10.1038/s41467-022-29009-w (PMC8964671; doi:10.1038/s41467-022-29009-w)
Supplement: Supplementary file 1 — Supplementary Information [file 41467_2022_29009_MOESM1_ESM.pdf]

Supplementary Information for "Large influence of atmospheric vapor pressure deficit on ecosystem production efficiency" by Lu et al.

Supplementary Information

Large influence of atmospheric vapor pressure deficit on ecosystem production efficiency

Haibo Lu[1], Zhangcai Qin[1], Shangrong Lin[1], Xiuzhi Chen[1], Baozhang Chen[2,3], Bin He[4], Jing Wei[1], Wenping Yuan[1*]

[1] School of Atmospheric Sciences, Southern Marine Science and Engineering Guangdong Laboratory (Zhuhai), Sun Yat-sen University, Zhuhai, Guangdong 519082, China

[2] School of Remote Sensing and Geomatics Engineering, Nanjing University of Information Science and Technology, Nanjing, Jiangsu 210044, China

[3] State Key Laboratory of Resource and Environmental Information System, Institute of Geographic Sciences and Natural Resources Research, Chinese Academy of Sciences, Beijing 100101, China

[4] State Key Laboratory of Earth Surface Processes and Resource Ecology, College of Global Change and Earth System Science, Beijing Normal University, Beijing 100875, China

Corresponding author: Wenping Yuan, yuanwp3@mail.sysu.edu.cn

Matters Arising from Liu, L. et al. Soil moisture dominates dryness stress on ecosystem production globally. *Nat. Commun.* **11**, 4892 (2020).

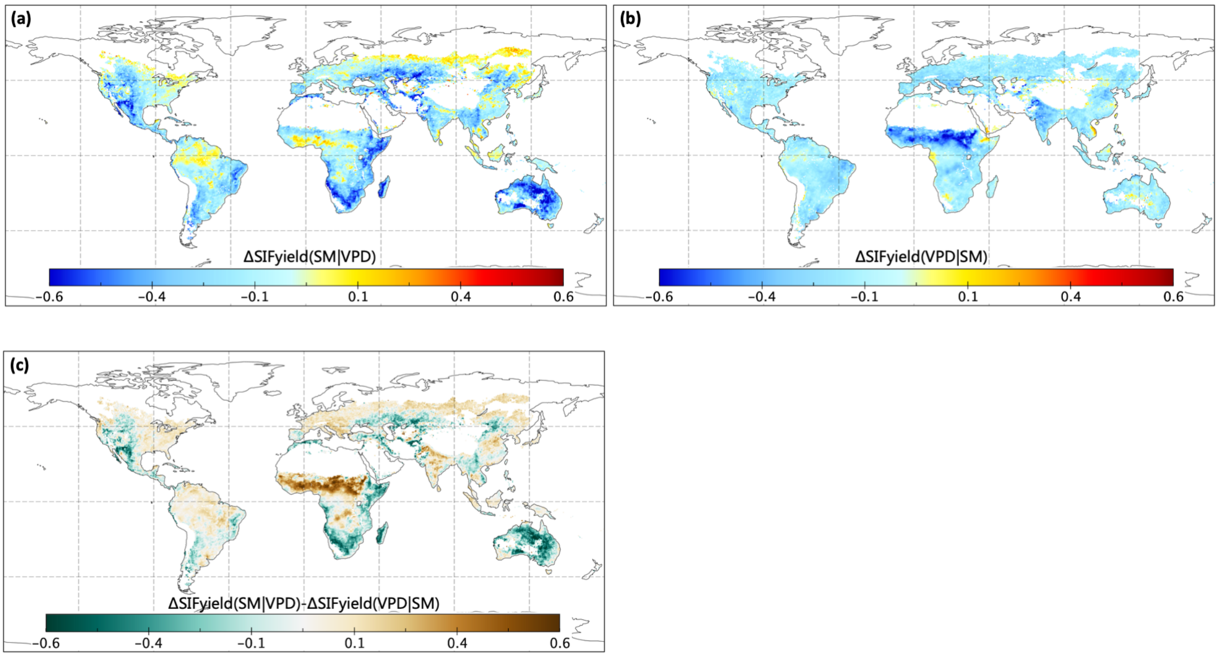

Supplementary Figure 1. The comparison on impacts of soil moisture (SM) and vapor pressure deficit (VPD) on fluorescence quantum yield (SIF_{yield}). (a) ΔSIF_{yield}(SM|VPD) indicates the changes of SIF_{yield} caused by soil moisture decrease. (b) ΔSIF_{yield}(VPD|SM) indicates the changes of SIF_{yield} caused by VPD increase. (c) Differences between ΔSIF_{yield}(SM|VPD) and ΔSIF_{yield}(VPD|SM). The positive values indicate larger impacts of VPD relative to SM in (c). Note, where ΔSIF_{yield}(SM|VPD)>0, the difference equals to ΔSIF_{yield}(VPD|SM) in (c); where ΔSIF_{yield}(VPD|SM)>0, the difference is ΔSIF_{yield}(SM|VPD); and where both are positive, the difference is not shown. For better comparability in space, the SIF_{yield} data time series was normalized by the average SIF_{yield} exceeding 90th percentile per pixel. The units refer to the fractions relative to average SIF_{yield} exceeding the 90th percentile in each grid cell.

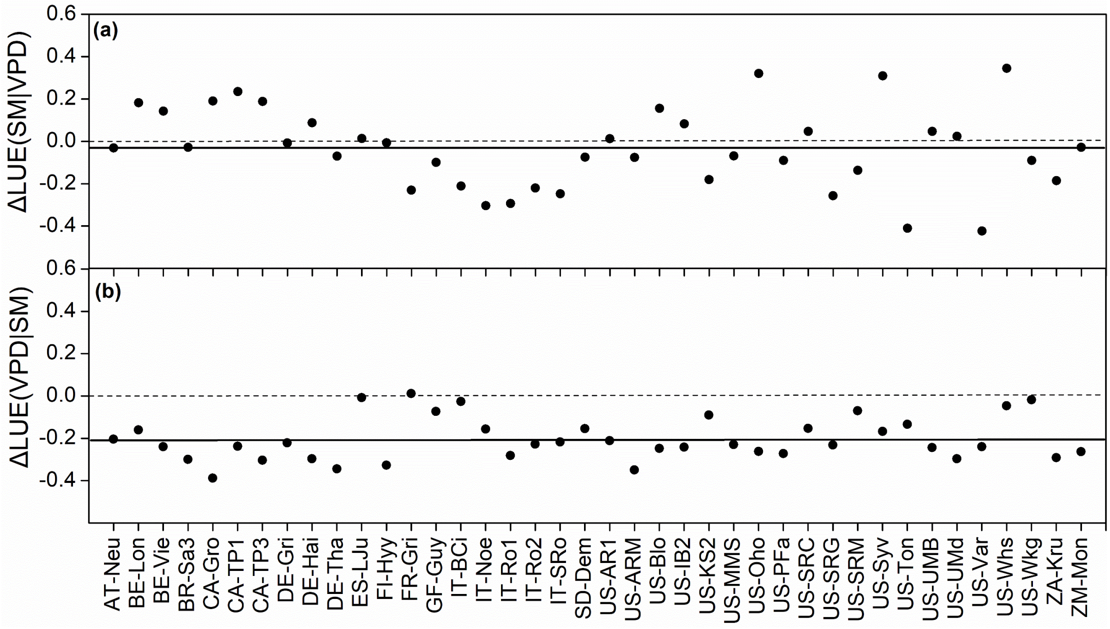

Supplementary Figure 2. The comparison on impacts of soil moisture (SM) and vapor pressure deficit (VPD) on light use efficiency (LUE) at 40 eddy covariance towers. (a) ΔLUE(SM|VPD) indicates the changes of LUE caused by soil moisture decrease. (b) ΔLUE(VPD|SM) indicates the changes of LUE caused by VPD increase. The dark solid lines in (a) and (b) indicate the mean ΔLUE(SM|VPD) and ΔLUE(VPD|SM) across all sites, respectively. For better comparability in space, the LUE data time series was normalized by the average LUE exceeding 90th percentile for each site. The units refer to the fractions relative to average LUE exceeding the 90th percentile at each site.

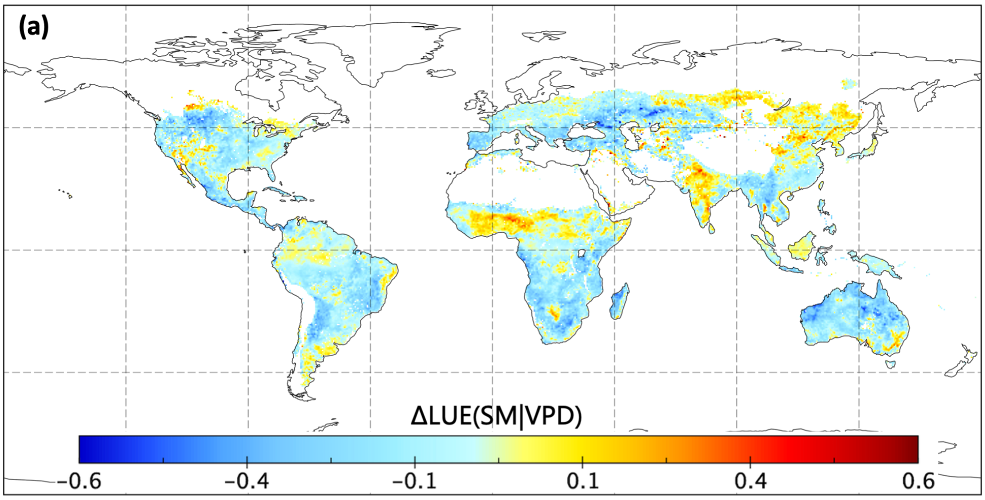

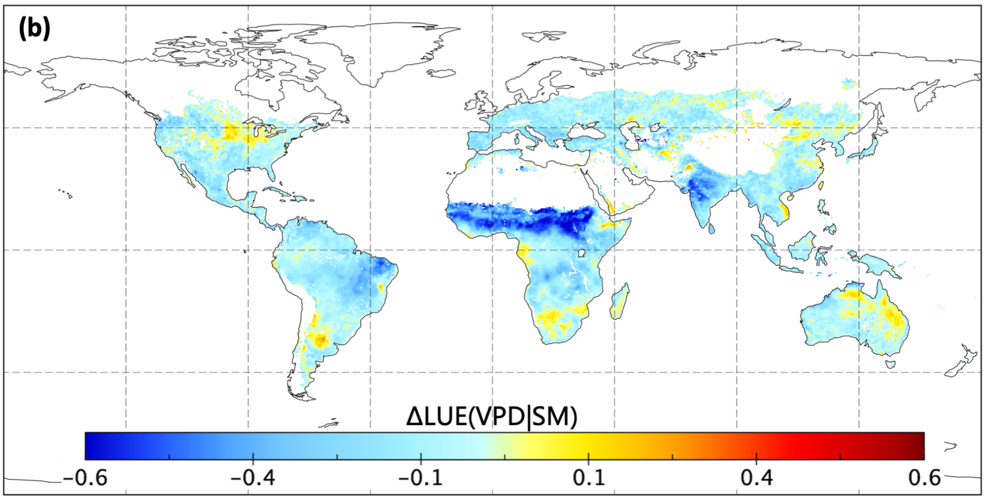

Supplementary Figure 3. The comparison on impacts of soil moisture (SM) and vapor pressure deficit (VPD) on light use efficiency (LUE) derived from FLUXCOM dataset globally. (a) ΔLUE(SM|VPD) indicates the changes of LUE caused by soil moisture decrease. (b) ΔLUE(VPD|SM) indicates the changes of LUE caused by VPD increase. For better comparability in space, the SIF_{yield} data time series were normalized by the average SIF_{yield} exceeding 90th percentile per pixel. The units refer to the fractions relative to average SIF_{yield} exceeding the 90th percentile in each grid cell.

Supplementary Table 1. List of eddy covariance sites used in this study.

Site	Latitude	Longitude	Type*	Study Period
AT-Neu	47.11°N	11.31°E	GRA	2002-2012
BE-Lon	50.55°N	4.75°E	CRO	2004-2014
BE-Vie	50.31°N	5.99°E	MF	1996-2014
BR-Sa3	3.02°S	54.97°W	EBF	2000-2004
CA-Gro	48.22°N	82.15°W	MF	2003-2014
CA-TP1	42.66°N	80.56°W	ENF	2002-2014
CA-TP3	42.71°N	80.35°W	ENF	2002-2014
DE-Gri	50.95°N	13.51°E	GRA	2004-2014
DE-Hai	51.08°N	10.45°E	DBF	2000-2012
DE-Tha	50.97°N	13.57°E	ENF	1996-2014
ES-LJu	36.93°N	2.75°W	SHR	2004-2013
FI-Hyy	61.85°N	24.30°E	ENF	1996-2014
FR-Gri	48.84°N	1.95°E	CRO	2004-2014
GF-Guy	5.29°N	52.92°W	EBF	2004-2014
IT-BCi	40.52°N	14.96°E	CRO	2004-2014
IT-Noe	40.61°N	8.15°E	SHR	2004-2014
IT-Ro1	42.41°N	11.93°E	DBF	2000-2008
IT-Ro2	42.39°N	11.92°E	DBF	2002-2012
IT-SRo	43.73°N	10.28°E	ENF	1999-2012
SD-Dem	13.28°N	30.48°E	SAV	2005-2009
US-AR1	36.43°N	99.42°W	GRA	2009-2012
US-ARM	36.61°N	97.49°W	CRO	2003-2012
US-Blo	38.90°N	120.63°W	ENF	1997-2007
US-IB2	41.84°N	88.24°W	GRA	2004-2013
US-KS2	28.61°N	80.67°W	SHR	2003-2006
US-MMS	39.32°N	86.41°W	DBF	1999-2014
US-Oho	41.55°N	83.84°W	DBF	2004-2013
US-PFa	45.95°N	90.27°W	MF	1995-2014
US-SRC	31.91°N	110.84°W	SHR	2008-2014
US-SRG	31.79°N	110.83°W	GRA	2008-2014
US-SRM	31.82°N	110.87°W	SAV	2004-2014
US-Syv	46.24°N	89.35°W	MF	2001-2014
US-Ton	38.43°N	120.97°W	SAV	2001-2014
US-UMB	45.56°N	84.71°W	DBF	2000-2014
US-UMd	45.56°N	84.70°W	DBF	2007-2014
US-Var	38.41°N	120.95°W	GRA	2000-2014
US-Whs	31.74°N	110.05°W	SHR	2007-2014
US-Wkg	31.74°N	109.94°W	GRA	2004-2014
ZA-Kru	25.02°S	31.50°E	SAV	2000-2013
ZM-Mon	15.44°S	23.25°E	DBF	2000-2009

*CRO: cropland; DBF: deciduous broadleaf forest; EBF: evergreen broadleaf forest; ENF: evergreen needleleaf forest; GRA: grassland; MF: mixed forest; SAV: savanna; SHR: shrubland.

Supplementary Table 2. List of datasets used in this study.

**Variables**	**Dataset name**	**Coverage, resolution**	**Time span**	**References and sources**
Solar-induced chlorophyll fluorescence	OCO2-CSIF	Global, 0.5°×0.5°; 4-day	2001 - 2016	Zhang, et al. [1] https://figshare.com/articles/CSIF/6387494
Gross primary production (GPP)	FLUXCOM	Global, 0.5°×0.5°; daily	1982 - 2013	Tramontana, et al. [2] http://www.fluxcom.org/CF-Download/
Estimated GPP based on eddy covariance measurements	FLUXNET2015	Site, daily	1995 - 2014	Pastorello, et al. [3] https://fluxnet.org/data/fluxnet2015-dataset/
Soil moisture; Air temperature; Dewpoint temperature; Air pressure	ERA-Interim	Global, 0.5°×0.5°; 6-hour	2001 - 2016	https://www.ecmwf.int/en/forecasts/datasets/reanalysis- datasets/era-interim
Photosynthetically active radiation (PAR)	MERRA2	Global, 0.5°×0.625°; 1-hour	1982 - 2016	https://disc.sci.gsfc.nasa.gov/datasets/M2T1NXLFO_5.12.4/summary?keywords=MERRA2_tavg1_2d_lfo_NX
Fraction of photosynthetically active radiation absorbed by plants (fPAR)	GLASS	Global, 0.5°×0.5°; 8-day	1982 - 2018	Xiao, et al. [4] http://www.glass.umd.edu/

Supplementary References

1 Zhang, Y., Joiner, J., Alemohammad, S. H., Zhou, S. & Gentine, P. A global spatially contiguous solar-induced fluorescence (CSIF) dataset using neural networks. *Biogeosciences* **15**, 5779-5800 (2018).

2 Tramontana, G. et al. Predicting carbon dioxide and energy fluxes across global FLUXNET sites with regression algorithms. *Biogeosciences* **13**, 4291-4313 (2016).

3 Pastorello, G. et al. The FLUXNET2015 dataset and the ONEFlux processing pipeline for eddy covariance data. *Sci. Data* **7**, 1-27 (2020).

4 Xiao, Z., Liang, S., Sun, R., Wang, J. & Jiang, B. Estimating the fraction of absorbed photosynthetically active radiation from the MODIS data based GLASS leaf area index product. *Remote Sens. Environ.* **171**, 105-117 (2015).
